# A Treatise on the Diseases of the Chest, and on Mediate Auscultation

**Published:** 1835-04-01

**Authors:** 


*A Treatise on the Diseases of the Chest, and on Mediate Auscultation.* By R. T. H. LAENNEC, M.D. *Translated from the latest French Edition, with copious Notes,* by JOHN FORBES, M.D., F.R.S. *Fourth Edition; with Plates.*—London, 1834. 8vo. pp. 675.

THE treatise of the immortal Laennec is so well known to the great proportion of readers, that it would seem almost a work of supererogation to set about a formal review of it at this period. Such, however, is not our intention. Dr. Forbes's translation, the first edition of which appeared many years ago, was the means of making auscultation known and employed in this country, although there is much reason to apprehend that prejudice still opposes its progress in many quarters. We have the best proof of the extent to which thoracic diseases, and their diagnosis, have interested the medical public, in the fact that the work at present before us is the fourth edition of Dr. Forbes's translation. A period of fifteen years has elapsed since the publication of Laennec's original treatise, and many improvements in auscultatory diagnosis, as well as other points connected with chest diseases, have taken place in that time: we purpose taking a review of these changes, as suggested to us by the very copious and valuable notes appended to the present edition.

Of late years, the original cylinder of Laennec has been turned and fined down into a delicate and portable tube, neatly tipped and inlaid with ivory: this modification, introduced by M. Piorry, is, we are glad to perceive, not approved by Dr. Forbes: it is faulty in having the conducting power of the wood impeded by screws and a thick cap of ivory, and he is of opinion that the one last used and recommended by Laennec is still the best, "with the only alteration of having the stopper made conical, in place of being rounded."

Within the last few years, the indefatigable researches of M. Piorry have brought percussion, as a means of diagnosis, very much into notice. M. Piorry employs percussion in a mediate manner: the following is our annotator's account of it.

"This improvement consists in interposing between the point of the fingers and the chest, a small plate of ivory on which the percussion is made; and from which circumstance, the inventor has, in imitation of Laennec, given the name of *Mediate* Percussion to his method. The ivory plate (which has received the name of *Pleximeter* or *Plessimeter*, from the words πλήσσω, *I strike*, or

πλῆξις, *percussion,* and μέτρον, *measure,*) is of a circular or ovoid shape, from an inch and a half to two inches in diameter, and about one sixth of an inch in thickness. It has either a raised edge or rim, or projecting handles, on its upper side, to permit its being held between the finger and thumb of the left hand, while it is struck with the right. In making use of this instrument, all that seems essential is to apply it accurately, closely, and consequently parallel to the surface. As in simple percussion, the blow may be made with one or more fingers, and must be rapidly executed, with the points but not the nails of the fingers; on this account the nails must be kept short. The pleximeter may be applied immediately on the skin or over some portion of the clothes; and, as in the case of the stethoscope, it is necessary on some parts to interpose a small pledget of lint or soft linen, to ensure its accurate apposition." (P. 23, *note.*)

The pain which is frequently attendant on immediate percussion, constitutes one of the principal objections to it, and is entirely obviated by M. Piorry's invention. Mediate percussion has also the advantage of enabling us to percuss in all parts of the thorax, and obviates many of the difficulties of percussing where the integuments of the chest are fat or anasarcous. M. Piorry considers that mediate percussion is much easier, and requires much fewer precautions, than the old method. "In direct percussion, we must never lose sight of the rule that the percussion must be made precisely in the same manner on the two opposite sides of the chest, to enable us to deduce safe conclusions from the resulting sounds." This precaution is unnecessary with the pleximeter, because we have here always the same flat, smooth surface whereon to strike, and an artificial wall everywhere, of equal density and elasticity.

Dr. Forbes mentions a more simple kind of mediate percussion, which is fully deserving the attention of the student.

"This consists of the substitution of one or two fingers of the left hand for the pleximeter, the *back* of the fingers being uppermost. This proceeding possesses several of the advantages of M. Piorry's method; and it has even some few over it, exclusive of its greater simplicity. In cases where there is considerable emaciation, M. Piorry's method is liable to mislead, unless the intercostal spaces are carefully filled with some soft material; as, without this precaution, the sound may be modified by the hollow existing between the plate and the skin. Direct percussion on the ribs, or the employment of the fingers as a pleximeter, is often, in such cases, preferable. If we are careful in applying the fingers so as to make them fit accurately into the natural depressions, and thus form one body, as it were, with the thoracic parietes, we are often enabled to use very forcible percussion without exciting pain, and also to elicit as definite sounds as by either of the other methods. This proceeding is free from another inconvenience which occasionally attaches to M. Piorry's method, especially in the hands of beginners. In the latter it sometimes happens that the loudness and sharpness of the primary sound arising from the contact of the two surfaces, are so considerable (particularly if the *nail* be used, which it ought never to be) as to drown, as it were, the secondary sound resulting from the modifying influence of the subjacent parts, from which modification it is that we form our judgment respecting the condition of those parts. When the fingers constitute the pleximeter, we have little or none of this immediate *clatter* when the blow is given." (P. 24, *note*.)

In looking through the chapters on the various signs ascertained by auscultation, we were particularly struck with the fact, which remarkably attests the extreme accuracy with which Laennec observed these sounds, that, since the publication of his last edition, all that he then stated has been confirmed in the most satisfactory manner; and the additions that have been made since that time are chiefly in exemplification or explanation of these sounds. Thus, under the head of Ægophony, that remarkable phenomenon of the voice which is heard in thoracic effusions, we find a note, by M. Meriadec Laennec, in which it is stated that "M. Reynaud has ascertained that, if an ægophonous patient lies on his belly, or leans forward, so as to bring the body into an almost horizontal position, not only does the ægophony disappear from the interscapular region, but is replaced by a bronchophony, of a greater or less intensity, according as the lung is sound or in a state of inflammation. In the latter case, as the ægophony vanishes, the crepitous rhonchus, or the bronchial respiration, reappears. From this M. Reynaud infers, that ægophony is merely a remote bronchophony,—that is to say, a bronchophony heard through a layer of fluid, of greater or less thickness." Such of our readers as may be familiar with Laennec's work will not fail to remember the experiments which he performed relative to this subject, as related at page 43 of the present edition. He applied a bladder, half filled with water, between the scapulæ of a young man, who presented a well-marked natural bronchophony in this point. "In this case," he adds, "it appeared to myself, and several persons present, that the voice, as transmitted through the liquid, became more acute, and also slightly tremulous, although less decidedly so than in real ægophony." From this it is obvious that Laennec's opinion as to the cause of ægophony was not very dissimilar to that lately expressed by M. Reynaud.

In speaking of the sound which is denominated *frottement ascendant et descendant*, or, according to the interpretation of Dr. Forbes, friction of ascent and descent, Laennec conjectured that, besides the interlobular emphysema, of which he considered it the pathognomonic sign, there was another lesion, which might give occasion to this sound of friction, viz. the existence of a cartilaginous, bony, tuberculous, or other indurated tumour, projecting from the surface of the lung. How far this conjecture was well founded, will appear from the observations of M. Reynaud, as detailed in the following interesting note of M. Meriadec Laennec.

"Subsequent observations have enabled M. Reynaud to establish the fact which Laennec only conjectured. The sound of friction is perceived in every case where the pleura is rough or uneven. It exists in pleurisy with little or no liquid effusion, and where the pleura is merely covered with a false membrane; and likewise in cases where the fluid is only in moderate quantity, and the free motion of the lung is not impeded by ancient adhesions. In this last case, when the lung, in certain positions of the body, rises above the level of the effusion, and rubs against the thoracic parietes, the sound of friction is heard immediately over this point. When the effusion becomes considerable, it disappears, and again returns when the fluid is lessened. In most cases the sound is perceptible by the application of the hand, as well as by auscultation: it may even be heard at some distance from the patient; and sometimes it is very perceptible to the latter. The sound of friction is not, it will now be perceived, exclusively confined to the case of pulmonary emphysema: it is met with in pleurisy, and may be regarded as a good sign, since it indicates that the effusion is not so great as to prevent the lung from being dilated, so as to reach the walls of the chest. Neither does it appear, as Laennec imagined, to be different in the two cases. I am even disposed to believe, that when it exists in emphysema, this affection is complicated with pleurisy. In confirmation of this, I may state, that in the notes of Laennec's own cases taken by myself, I find, almost always, this complication expressly named, where the sound of friction is recorded. The same observation applies to M. Reynaud's third case (Journ. Hebd. No. 65); and even Andral bears testimony to the accuracy of M. Reynaud's views. (Clin. Med. t. ii. p. 613, 2d Ed.)

"The foregoing pathological facts led M. Reynaud to examine whether there might not exist an habitual friction between the pulmonary and costal pleura in the state of health. And this seemed established by *a priori* considerations, namely, by the invariable formation of an accidental serous tissue wherever a false joint or accidental movement is established, and by the obliteration of the articular serous cavities on the abolition of all motion of the parts. With the view of proving the fact, Mr. Reynaud made an experiment on a living animal, and believed that he could distinctly perceive the motion of the lungs against the ribs during inspiration and expiration. In the state of health, the sound produced by this friction of the parts is not perceptible, owing to the slippery smoothness of the two membranes, or is confounded with that of the respiration; but when the natural condition of the parts is altered by inflammation or any other cause, it then becomes manifest. (See Journ. Hebdom. de Med. No. 65.)—*M. L.*" (P. 58, *note.*)

For the amusement, more than for the instruction, of our readers, we transcribe the following note by the translator, which will be found towards the end of the section on Hooping-cough.

"The following is a brief synoptical view of the principal opinions promulgated by the moderns concerning the seat and nature of the hooping-cough, with the names of their chief supporters. It is proper, however, to remark, that several of the writers included under the same head, although agreeing generally as to the nature of the disease, sometimes advocate considerable and peculiar modifications of the common doctrine.

"1. A nervous disease, according to the common acceptation of that term.—Cullen; Böhme; Guibert.

"2. An idiopathic affection of the pulmonic and diaphragmatic nerves.—Hufeland; Jahn; Lobenstein; Albers; Wendt; Paldamus.

"3. A nervous affection of the lungs, from sympathy with other organs but chiefly with the stomach and bowels.—Stoll; Butter; Waldschmidt; Chambon; Danz(?).

"4. A catarrh of the lungs and stomach. 'Affection pneumo-gastrique pituiteuse.'—Tourtelle.

"5. The same, but conjoined with a spasmodic affection of the glottis and diaphragm.—Gardien; Millot.

"6. Primary affection of the brain, exciting spasmodic affection of the respiratory apparatus.—Leroy; Boisseau; Webster; Otto; Begin.

"7. Inflammation of the larynx and glottis.—Astruc; Dawson.

"8. Primary bronchitis, or pulmonary catarrh, inducing directly spasm of some part of the respiratory apparatus.—Darwin; Watt; Marcus; Laennec; Broussais; Guersent; Dewees; Fourcade-Prunel; Duges.

"9. Primary bronchitis inducing cephilic irritation, and this in its turn exciting the spasmodic affection of the respiratory organs.—Desruelles.

"10. Insects irritating the bronchial membrane.—Rosenstein; Linnæus." (P. 93, *note.*)

So much for the certainty of our knowledge of those diseases which leave no constant traces of morbid structure!

We cannot pass by the chapter on Pulmonary Apoplexy, without one or two remarks. This name was given by Laennec to that morbid condition of the pulmonary structure which is consequent on extravasation of blood into it. "From its exact resemblance," he says, "to the effusion that takes place in the brain in apoplexy, I have thought the name Pulmonary apoplexy very applicable to it, as it resembles, in every respect, the cerebral hemorrhage commonly termed apoplexy." There are some who, in their hypercriticism, object to this term, inasmuch as there are no *symptoms* analogous to those of cerebral apoplexy; yet we have ever thought that the suddenness which characterizes the hæmoptoic seizure, and the prostration which follows, are by no means inconsistent with the designation "apoplexy:" and when we add to this the similarity in organic changes, we cannot reject a term which so completely associates this disease with one which in its essence is precisely similar. This similarity, or "perfect analogy," has lately been completely established in a thesis by M. Roupel (*Recherches sur les Hemorrhagies*, Paris, 1827,) and in the recent treatise on Apoplexy, by Cruveilhier, (*Dict. de Med. et Chir.*, art. *Apoplexie.*)

"We may observe" (says Meriadec Laennec,) "in the lungs, as in the brain, and indeed in most of the other organs, the three forms of hæmorrhage, viz.—1. *The blood-stroke*, (*coup-de-sang*,) an instantaneous and universal congestion without any escape of blood from the vessels; of this form the lungs offer an example in the case of asphyxia, in which the pulmonary tissue, without losing its wonted crepitation on being handled, is coloured of a dark red hue, and pours out, when incised, a profusion of fluid black blood: 2. *Apoplexy*, properly so called, such as is described in the text, and varying from simple infiltration to the largest coagula of blood, with rupture of the vessels and laceration of the organ; 3. Slow hæmorrhagic infiltration or splenisation, in which the tissue of one whole lung or one lobe slowly and progressively penetrated by blood, assumes a darkish red colour, and becomes smooth, heavy, hemogeneous, and friable as the spleen, with the organization of which it presents a resemblance more or less close. This last variety of pulmonary apoplexy is common in old persons who have been long confined to bed in one posture. It is also observed after diseases of an adynamic kind, whether acute or chronic. The splenised portions are sometimes softened partially or totally, being converted into a sort of blackish paste, which we might mistake for the effect of putrefaction. In some cases these portions are intermixed with spots of the inflammatory hepatization, recognized by their red or yellowish colour, and which contrast well with the dark ground of the general mass.—(*M. L.*)" (P. 172, *note.*)

It is a doctrine now very commonly received, that pneumonia most commonly attacks the lower lobes of the lung; and upon this Laennec founded one of his strongest arguments against the Broussaian doctrine of the inflammatory origin of tubercles, which, it is universally admitted, have a marked predilection for the upper lobes. It does not appear, however, that the subsequent experience of MM. Andral, Chomel, and Frank, which has been referred to in a note by our translator, very fully confirms this opinion of Laennec. Andral found that, out of 88 cases of pneumonia, the inferior lobe was affected in 47, the upper lobe in 30, and the whole lung in 11; and Chomel states *(Dict. de Med.)* that, in 59 cases, the upper lobes were affected in *thirteen*, the lower in 11, and the whole of one lung in 31, the posterior parts in two, and the middle in one; and Frank says, "Frequentius forte *superiores* pulmonum lobos inflammatos deteximus."

The greater frequency, however, of the occurrence of pneumonia on the right side than on the left, as stated by Laennec, and before him by Morgagni and many others, has received the fullest confirmation. Andral found that, out of 204 cases of pneumonia, the right lung was affected in 121 cases, the left in 58, and both in 25; and Chomel says, that, in 59 dissections, the right lung was affected in 28, the left in 15, and both in 16. Lambard states that, of 868 cases of pneumonia, 413 were on the right side, 260 on the left, and 195 on both. Dr. Forbes adds, "The general result of these cases is, that, out of a total of 1131 cases, the right side was affected in 562, the left in 333, and both in 236. Taking these results in round numbers, and approximating and assuming them to give a fair view of the general habitudes of the disease, we would say that, out of every ten cases of pneumonia, we might expect five, or one half, to be confined to the right side, three to the left, and two to extend to both."

Of all the stethoscopic signs, Laennec regarded the *rhonchus crepitans* as the most practically useful, inasmuch as it points out, in its very earliest stage, one of the most common and most severe diseases. In this, we have no doubt, every practical physician must agree with him; yet it is to be remembered, that pneumonia may be present, and yet the *rhonchus crepitans* absent. This is Chomel's opinion, who likewise states that this *rhonchus* may be present in cases where the existence of inflammation is extremely doubtful. Cruveilhier likewise greatly undervalues this sound as a sign in pneumonia, especially when compared with those of bronchophony and tubary respiration. M. Meriadec Laennec makes the following judicious remarks relative to the doubts that have been expressed by some as to the value of the *rhonchus crepitans.*

"The doubts of men so eminent as Andral, Chomel, and Cruveilhier, demand from us some investigation whether there may not exist certain circumstances calculated to produce mistake respecting the value of the crepitous rhonchus. In the first place, it is possible that the obscure mucous rhonchus may be mistaken for it, the more so because the two are nearly allied both in their cause and character, and are, in truth, not easily discriminated by the most experienced. Secondly, the crepitous rhonchus may have been really heard during life, and yet no trace of inflammation be found after death, because this has taken place during the stage of resolution of the pneumonia. Thirdly, pneumonia may actually be present and yet the rhonchus be wanting, from the circumstance that the respiration is too feeble to force the air into the engorged vesicles, owing to the age of the patient, or the debility produced by preceding disease. For an analogous reason, of an opposite kind, the crepitous rhonchus may be sometimes perceptible when there is no pneumonia, in the case of children, the extreme power of whose respiration may excite in their diminutive bronchial ramifications a mere mucous rhonchus with bubbles as small as those which constitute the crepitous.—(*M. L.*)" (P. 195, *note.*)

The chapter on Phthisis Pulmonalis is enriched by the addition of several notes from the pen of the French editor, as well as of the English translator. Since Laennec's time, morbid anatomists have been much occupied by researches into the nature and mode of development of tubercles, especially as they appear in the lungs. We proceed to inquire whether any, and what, advances have been made towards the acquisition of correct views respecting the pathology of this disease.

According to Laennec, tubercle first appears as a miliary granulation, supposed by Bayle to be a production different from internal; an opinion likewise maintained by Andral and Chomel. The second stage was that of the miliary, the third the crude, and lastly the encysted, tubercle. Laennec, it will be remembered, also thought that miliary tubercles were accidental productions, possessing a proper vitality, and growing by intussusception. When the tubercle has arrived at the stage of crudity, its next tendency is to become soft and fluid. "The process," says Laennec, "begins in the centre of each mass, and gradually increases the tuberculous matter, becoming daily softer and moister, cheesy, at least unctuous to the touch, like soft cheese, and finally acquires the viscidity and fluidity of pus." These views have, however, been objected to by several pathologists, most of whom regard tubercle as the result of a morbid secretion, which may be excited or promoted by inflammatory action, but occurs more commonly in consequence of a general predisposition, congenital or acquired; and which predisposition seems, in its turn, to be the result, at least most frequently, of an altered condition of the fluids. The original opinion of Bayle, respecting the distinctness of pulmonary granulations from tuberculous matter, is maintained by those pathologists, especially Andral, in opposition to that of Laennec, who considered them to be related to each other, as green fruit is to ripe fruit. (P. 255.) Andral regards these granulations as the result of very limited pneumonias, and does not admit the existence of the grey tubercle of Laennec; the change of structure to which that name has been applied being a mere variety of chronic inflammation, within which true tubercle is frequently developed, though by no means as a necessary consequence.

M. Rochoux, following some observations originally made by Dalmazzone, concludes that tubercle is neither an accidental production nor a secreted matter, but a degeneration or transformation of a healthy tissue into a morbid one; and Dr. Baron, whose opinion is supported by Dupuy, of the Ecole Vétérinaire at Alfort, contends that all tubercles are, in their origin, transparent vesicles or hydatids; upon which M. Meriadec Laennec remarks, "This opinion is evidently that of a man little versed in the researches of pathological anatomy, and more conversant with slaughter-houses than dissecting rooms, who has not been able to discriminate the distinctive progress of each of two morbid alterations frequently coexisting." (P. 258.)

Dr. Carswell coincides with Andral and Cruveilhier as to the formation of tubercle by secretion. He offers an explanation of the apparent commencement of softening in the centre, by supposing that the tuberculous matter is deposited from the mucous lining of the bronchus, and encloses in its centre some of the mucus secreted by that membrane. When this is cut, the mucus appears as a soft point in the centre of the tubercle, which Laennec regarded as the commencement of the softening process. In some instances no such appearance is visible, and in these cases the bronchi or air-cells must have been completely filled with the tuberculous deposit. Dr. Carswell questions the correctness of the term "*encysted*," the imaginary cyst being nothing more than the walls of the air-cell in which the tubercle is deposited.

It may well be doubted, then, whether any addition has been made to our knowledge of the anatomy of tubercle subsequently to the time of Bayle and Laennec. That which most baffles our efforts is the primary origin of these formidable morbid productions; and although, in reasoning analogically, there seems to be strong reason to consider the theory of the *secretion* of tubercle the correct one, yet we want demonstrative evidence of the fact, and probably shall ever continue to want it, as regards this as well as all the other secret operations of nature. Although much has been added to the statistical details respecting phthisis, we see no progress towards such a mode of treatment as will eradicate the disease: the curability of it, however, is now fully established and admitted, and we are thus in some degree encouraged to proceed in our efforts to arrive at some more powerful method of therapeutics.

We pass over the section on Asthma and Diseases of the Pleura, as they present little of novelty; not omitting, however, to recommend to the perusal of our readers some very good remarks, by Dr. Forbes, on the treatment of asthma, in a note of some extent, in which he has related and compared the various plans of treatment proposed or adopted for the cure of that disease.

We now come to that portion of the work which was confessedly left by Laennec himself in the least perfect state: we mean the application of auscultation to the diagnosis of diseases of the heart and great vessels. It is well known that even the sounds afforded by the normal action of the heart's cavities can hardly be said to be perfectly understood. The deference naturally paid to so accurate an observer as Laennec led physicians too much to take for granted the correctness of the statement made by him respecting the heart's action. However, it was ere long discovered that his hypothesis was inadequate to guide the physician in his diagnosis of heart-disease, and numerous researches were undertaken in support of various theories. There are but two sounds audible, the one rapidly follows the other, and then a marked interval of repose is manifest. One of these sounds is synchronous with the impulse of the heart against the parietes of the chest: these, then, are the phenomena which demand explanation. The first sound which is heard after the interval of repose is that which accompanies the impulse, and this is supposed by Laennec, Hope, Rouanet, and Carlile, to be caused by the ventricular *systole.* Dr. Hope, ascribing the generations of the sound immediately to the collision of the particles of fluid in the ventricles; M. Rouanet, to the closing of the mitral and tricuspid valves; and Mr. Carlile, to the rush of blood into the arteries, consequent upon the systole of the ventricle. But by Corrigan, Pigeaux, and Magendie, it is thought to occur during the *diastole* of the ventricle; the two former of these writers attributing it to the rush of blood into the ventricle, impelled by the auricle; and the last, to impulsion of the apex of the heart against the chest, by the ventricular diastole. The second sound is attributed by Laennec to the systole of the auricles, in which opinion he stands alone; by Hope, to the *diastole* of the ventricles; by Magendie, to the impulsion of the *base* of the heart against the walls of the chest, by the *systole* of the ventricles; and in this view he coincides with Corrigan as to the coincidence of the second sound and systole. Pigeaux ascribes the second sound to the collision of the blood against the walls of the aorta and pulmonary artery; and Carlile, to the reaction of the blood in the arteries on the semilunar valves, at the moment of the ventricular diastole; but Dr. David Williams thinks it is caused by the flapping open of the auriculo-ventricular valves against the side of the ventricle.

Such is a brief statement of the diversity of opinion respecting the indications afforded by the phenomena of the heart's action, which, we think, is sufficient to show that the question is still *sub judice.* It will be seen, however, that the greatest number of observers are in favour of the isochronism of the first sound and systole of the ventricles, although these writers differ in their methods of explaining in what way that sound is generated. The same may be said of the second sound, as occurring at the moment of the diastole of the ventricles. The arguments for and against these several theories will be found in a very useful note, at p. 511 et seq., taken by Dr. Forbes from Dr. Williams' work on Ascultation: we can recommend the perusal of it to the reader who desires to consider the subject fairly and philosophically.

The following note, by M. Meriadec Laennec, to be found at the conclusion of the chapter on Rupture of the Heart, contains an interesting account of a lesion which had escaped even the penetration of Laennec.

"*Apoplexy of the heart*, an affection which I am surprised has not been mentioned by Laennec, and of which several examples have been recently published by M. Cruveilhier, (*Anat. Path.* 3. Liv. *Par.* 1829,) appears to be, much more frequently than inflammation, the cause of rupture of the heart. This lesion has hitherto been observed only in the walls of the left ventricle when in a state of hypertrophy. Here, as in the case of other muscles, the boundaries of the apoplectic deposit are formed partly by the muscular fibres ruptured and partly by their simple displacement. When quite recent, these deposits contain merely coagulated black blood; when they have existed some days, their walls are of a blackish red which penetrates to a greater or less depth, and we can distinguish some shreds of muscular fibres amid the blood; still later, the contained fluid assumes the colour of wine-lees, and appears as if formed of an admixture of blood and pus; and, at last, it becomes entirely purulent, and the walls of the abscess are lined by false membranes. M. Rousset, whose thesis I had occasion to notice in the chapter on Pulmonary Apoplexy, (*Rech. Anat. sur les Hemorrhagies, Par.* 1827,) has recorded a very fine case of muscular apoplexy, nearly universal, in which the heart was the site of three deposits, in the various stages just enumerated. These sanguineous or puro-sanguineous depositions in the walls of the heart, usually terminate in perforation either inwards into the cavity of the ventricle, or outwards into the pericardium. In the latter case their rupture is almost always immediately fatal. In the former case, the cavity becomes filled with the ventricular blood, and eventually the remaining exterior wall of the abscess being distended, gives rise, in all probability, according to the ingenious explanation of M. Cruveilhier (*Dict. de Méd. Pract.* t. iii. Art. *Apoplexie*) to those partial dilatations of the heart, described by M. Breschet under the name of false consecutive aneurisms of the heart, and of which two cases by M. Berard were noticed in Chap. VII. of the present book. M. Reynaud, however, in a notice of a particular kind of aneurism of the heart, (*Journ. Hebd. de Méd.* t. ii. p. 363.) has attempted to show that these excavations in the walls of the left ventricle are sometimes, in reality, the result of a partial dilatation, they having been observed to be lined (acccording to him) with a membrane continuous with that of the natural lining of the ventricle. But in the cases adduced in support of this opinion by M. Reynaud, it is observable that the lining membrane of the aneurismal sac was thickened around the orifice of communication between it and the cavity of the ventricle: a circumstance which alone suffices, in my judgment, to prove, that the membrane lining the aneurismal sac was not a continuation of that of the ventricles, but an old adventitious membrane analogous to those which line fistulæ in ano, and are continuous with the mucous membrane of the rectum. In this point of view, then, the case of M. Reynaud differs in no essential respect from those of MM. Cruveilhier and Rousset, being merely an example of the manner in which the apoplectic abscess of the heart became cicatrized. All the cases of rupture of the heart hitherto published, appear to me to confirm the opinion that cardiac apoplexy is the most common cause of them. It is proper to state, however, that M. Rachoux, who appears to have carefully examined the same facts, gives a preference to the explanation of the phenomenon by means of a softening of the heart; (*Dict. de Méd.* t. xvii. Art. *Rupture;*) and yet when we consider that in the opinion of this gentleman, every apoplexy is preceded by softening, we may, after all, consider his opinion as not being essentially different from that of M. Cruveilhier. Respecting the opinion of M. Cruveilhier, that apoplexy never affects the walls of the right ventricle, and that when rupture of them takes place, it depends on an atrophy, a fatty degeneration, or a gelatinous softening of the heart, I am not prepared to say how far it is well or ill founded. The recorded facts appear to justify it no farther than by this consideration, that the rupture of this ventricle being infinitely more rare than that of the left, it seems to indicate a different cause.—(*M. L.*)" (P. 581, *note.*)

The seat of rupture of the heart has thus been stated by Dr. Forbes, from an examination of fifty-seven cases, collected from various sources.

| Left Vent. | Right Vent. | Both Vent. | Right Aur. | Left Aur. |
|---|---|---|---|---|
| 32 | 13 | 3 | 7 | 7 |

On the subject of the abnormal sounds, whether existing in the heart or great vessels, the reader will find all the most modern opinions specified in the notes attached to the fifth chapter of the third part of the work, either from the pen of Meriadec Laennec, or of our English translator; and indeed we would say, that it is to this part of the work that the most interesting and valuable additions have been made.

Of this translation generally, and this edition in particular, we cannot but speak in terms of commendation: we know not that, had we ourselves undertaken the work, we should have dared to take such liberties with the original as Dr. Forbes has done, holding as sacred, as we do, all that comes from such a man as Laennec, and regarding him as the founder of the science of auscultation. He laid the foundation, and reared the edifice; and, although his short span of life did not permit him to see its full completion, yet such is the solidity of the substratum on which it rested, and such the excellence of the workmanship, that it may be safely said to bid defiance to the inroads of time.

